# 3D corrective osteotomy of malunited upper limb fractures

**DOI:** 10.1186/1753-6561-9-S3-A97

**Published:** 2015-05-19

**Authors:** Tsuyoshi Murase

**Affiliations:** 1 Department of Orthopaedics, Osaka University Graduate School of Medicine, Suita, 565-0871 Japan

## Introduction

Corrective osteotomy for malunited fractures of the upper extremity remains a challenging procedure. Anatomical correction is the key to good clinical outcome after corrective osteotomy for malunited fractures, especially in cases of the upper extremity. To make a precise deformity correction in malunion, we have developed a computer-assisted system for corrective surgery, including a three-dimensional (3-D) program and a patient specific instrument (PMI), and investigated the results of corrective surgery for malunited fractures of the distal radius (MFRD), forearm diaphyses (MFFD) and distal humerus (cubitus varus deformity: CVD) with the use of this technology.

## Materials and methods

Ten MFRD, 20 MFFD and 30 CVD were enrolled in this study. Both upper extremities including the whole affected bone were scanned with a CT machine and 3-D bone models were constructed on the computer. Then, the deformity of the affected bone was evaluated by superimposing it with the goal model (e.g., the mirror image of a contralateral normal bone), which can be further determined in terms of rotation around and translation along the 3-D deformity axis using the screw displacement axis technique [1]. With this information, the most appropriate corrective osteotomy was simulated, which could be a closing wedge osteotomy, opening wedge osteotomy or rotational osteotomy. Then, the PMI was designed and manufactured by a 3-D printer with medical grade resin. The PMI has a shape that exactly fits to the bone surface and guides the planned osteotomy and insertion of Kirschner wires that indicated the amount of correction. During the operation, corrective osteotomy was performed with the use of the PMI followed by internal fixation applied with plates and screws (MFRD, MFFD and adult CVD) or with Kirschner wires (pediatric CVD).

## Results

Primary bone union was achieved in 56 patients and secondary bone union after additional intervention in the remaining 4. All radiographic parameters and ranges of adjacent joint motion were almost normalized after surgery. Improvement was observed even for longstanding forearm deformity up to 96 months after the initial injury. Preoperative pain disappeared or reduced in most of the cases.

## Discussion

Computer-assisted osteotomy can provide excellent radiographic and clinical outcome for the treatment of malunited fractures of the upper extremity.

For malunited distal radius fracture, a combination of 3-D computer simulation and a volar locking plate provides a simple, systematic and accurate corrective surgery [2].

For malunited diaphyseal forearm fractures, achievement of axial alignment, and restoration of normal length of both bones are considered to be prerequisites for a clinically good outcome. However, malunion with complex 3-D deformities of both forearm bones is difficult to assess accurately by plain radiography. In addition, it is a demanding procedure to reduce two parallel rotating bones while maintaining the congruity of the adjacent joints by corrective surgery. Computer simulation and use of PMI help us to achieve the highly demanding procedure with relatively simple surgical technique [3]3.

For the cubitus varus deformity after supracondylar fracture that classically includes varus, extension, and internal rotation deformity, no reliable surgical method for 3-D corrective osteotomy has been established. Our method using a PMI designed on the basis of 3-D computer simulation realized simultaneous correction of the three deformity components [4,5].
